# The methods for inserting lumbar bicortical pedicle screws from the anatomical perspective of the prevertebral great vessels

**DOI:** 10.1186/s12891-019-2756-0

**Published:** 2019-08-17

**Authors:** Liehua Liu, Haoming Wang, Jiangang Wang, Qian Wang, Shiming Cheng, Ying Li, Weidong Jin, Zili Wang, Qiang Zhou

**Affiliations:** 1 Department of Spine Surgery, The Third Affiliated Hospital of Chongqing Medical University (Gener Hospital), 1 Shuanghu Branch Road, Yubei District, Chongqing, 401120 China; 2 Department of Spinal Surgery, General Hospital of Ningxia Medical University, Yinchuan, 750004 Ningxia Hui Autonomous Region China; 3 Department of Orthopedics, Three Gorges Central Hospital, Chongqing, 404000 China; 4 Department of Orthopedics, No. 13 People’s Hospital of Chongqing, Chongqing, 400053 China; 5 Hillsborough Community College, Tampa, FL 33614 USA; 6 Department of Orthopedics, Chongqing Dongnan Hospital, Chongqing, 401336 China; 7 Department of Radiology, Southwest Hospital, Army Medical University, Chongqing, 400038 China

**Keywords:** Bicortical pedicle screw, Vessel anatomy, Computed tomography angiography, Osteoporosis

## Abstract

**Background:**

At present, bicortical pedicle screws (BPSs) are not used clinically because they carry the potential risk of damaging the prevertebral great vessels (PGVs). The authors observed the anatomical relationship between the PGVs and simulated BPSs at different transverse screw angles (TSAs), exploring the insertion method of the BPS.

**Methods:**

Computed tomography angiography (CTA) images from 65 adults were collected. A total of 4–5 TSAs of the BPSs were simulated on the left and right sides of L1-L5 (L1-L3: 0°, 5°, 10°, 15°; L4-L5: 0°, 5°, 10°, 15°, 20°). There were three types of distances from the anterior vertebral cortex (AVC) to the PGVs (D_AVC-PGV_); D_AVC-PGV < 0.50 cm_, D_AVC-PGV ≥ 0.50 cm_, and D_AVC-PGV↑_; these distances represented close, distant, and noncontact PGV, respectively.

**Results:**

The ratio of every type of PGV was calculated, and the appropriate TSA of the BPS was recommended. In L1, the recommended left TSA of the BPS was 0°, and the ratio of the close PGV was 7.69%, while the recommended right TSA was 0°-10°, and the ratio of the close PGV was 1.54–4.62%. In L2, the recommended left TSA of the BPS was 0° and the ratio of the close PGV was 1.54%, while the recommended right TSA was 0°-15° and the ratio of the close PGV was 3.08–9.23%. In L3, the recommended left TSA was 0°-5°, and the ratio of the close PGV was 1.54–4.62%. In L4, the recommended left TSA was 0°, and the ratio of the close PGV was 4.62%. BPS use was not recommended on the right side of either L3 or L4 or on the either side of L5.

**Conclusions:**

From the anatomical perspective of the PGVs, BPSs were not suitable for insertion into every lumbar vertebra. Furthermore, the recommended methods for inserting BPSs were different in L1-L4.

## Background

As the population ages, the number of patients with osteoporosis is increasing [1, 2]. Many lumbar fractures caused by osteoporosis and geriatric lumbar degenerative diseases often require pedicle screw fixation [3, 4]. Due to the decreased holding force of osteoporotic bone, pedicle screws are prone to loosening or breaking and can result in failure of internal fixation [5, 6]. To enhance the stability of pedicle screw fixation in patients with osteoporosis, we studied the pedicle screw fixation methods that are described in detail below.

First, the use of bone cement to strengthen the fixation of pedicle screws has been successful in a clinical setting [7, 8]. However, the high temperature required for cement curing can kill the surrounding bone cells, resulting in bone absorption and loosening of the bone-cement interface [9]. In addition, there may be dangerous complications, such as bone cement leakage and pulmonary embolism [10], and revision surgery or infection after a cement screw is placed can be very difficult [11]. Second, some researchers have improved the design of pedicle screws through the creation of expansive pedicle screws that have certain mechanical properties [12, 13]. However, one study found little to no effect of screw size on the fixed stiffness of osteoporotic bone [14]. Finally, improvements have been made in screw insertion techniques, including the adjustment of screw orientation and length. Increasing the depth of screw placement increases the holding force of the pedicle screw [15]. When a screw breaks through the anterior cortex of the vertebral body to form a bicortical pedicle screw (BPS), the pullout strength of the screw is increased from 20 to 50% [11, 16]. Zhuang et al. [17] confirmed that the mechanical properties and early screw loosening of bone cement screws were not better than those of the BPSs. Because the abdominal aorta, inferior vena cava, and iliac vessels are located in front of the lumbar spine, a BPS that breaks through the anterior cortex of the lumbar vertebral body can potentially 0damage the prevertebral great vessels (PGVs) [18]. To the best of our knowledge, there have been no reports on the anatomy of the PGVs and their association with BPSs.

Therefore, we used computed tomography angiography (CTA) to observe the anatomical relationship between the PGVs and simulated BPSs with different transverse screw angles (TSAs), exploring the insertion method of the BPS.

## Materials and methods

### General information

From December 1, 2017, to May 31, 2018, CTA images from 65 adult patients with urological diseases were collected. All samples were from patients who had no lumbar deformity, no spondylolisthesis, no PGV vasculopathy, and no history of retroperitoneal surgery or spinal surgery. All methods of this experiment were in accordance with the relevant guidelines and regulations of the Helsinki Declaration. All experimental protocols were approved by the Research Ethics Committee of the General Hospital of Ningxia Medical University. Written informed consent was obtained from all the enrolled participants.

### Methods

A Somatom Definition dual-source spiral CT (SIEMENS Corporation, Munich, Germany) scanner was used to scan the abdomen, including the T12-S1 vertebral bodies. The scanning thickness was 5 mm, the pitch was 1.15 mm, and the reconstructed thickness was 1 mm, with an overlap of 30%. The contrast agent (Omnipaque) was injected mid-intravenously from the right elbow with a dose of 100 ml (100 ml: 37 g I) and an injection rate of 4 ml / s. The high-pressure syringe automatically triggers the scan. The scanning time of the arterial phase was 25 s–30 s, and that of the venous phase was 60 s–70 s. All images were subjected to maximum intensity projection, volume rendering techniques, and multiplanar reformation to clearly display the PGVs. The postprocessing workstation of the SIEMENS dual-source spiral CT was used to observe the anatomical relationship between the PGVs and the anterior lumbar cortex.

**Fig. 1:**
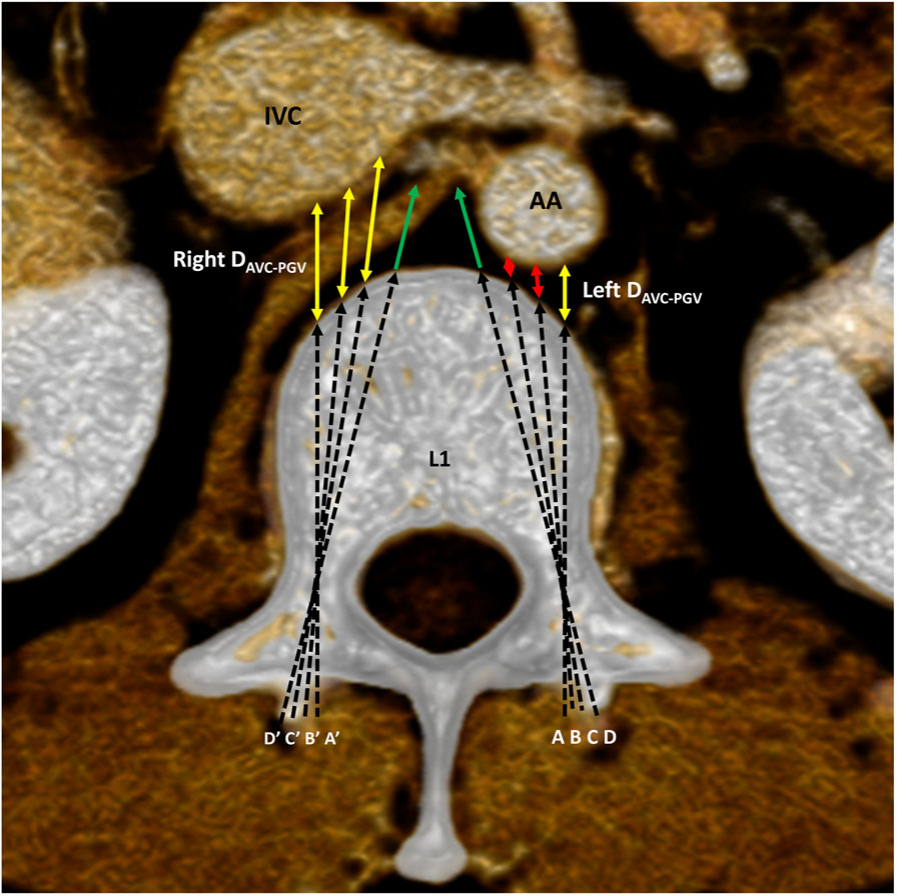
D_AVC-PGV_ at 0°, 5°, 10°, and 15° in L1. The cross-section of L1 was passed through the widest plane of the bilateral pedicles and was parallel to the superior endplate. A, B, C, and D represent the left TSA of the BPS at 0°, 5°, 10°, and 15°, respectively; A’, B′, C′, and D’ represent the right TSA of the BPS at 0°, 5°, 10°, and 15°, respectively. D_AVC-PGV_ represents the distance from the AVC to the PGV. The green single arrow lines represent the extent of the BPS that did not intersect the PGV; it was expressed as “↑”. The red double arrow solid lines represent D_AVC-PGV < 0.50 cm_, and the yellow double arrow solid lines represent D_AVC-PGV ≥ 0.50 cm_. The left D_AVC-PGV_ was 0.63 cm, 0.34 cm, 0.21 cm and ↑ at 0°, 5°, 10°, and 15°, respectively. The right D_AVC-PGV_ was 1.34 cm, 1.37 cm, 1.51 cm and ↑ at 0°, 5°, 10°, and 15°, respectively. AA: Abdominal aorta. IVC: Inferior vena cava

First, the cross-section of each lumbar vertebral body was selected and passed through the widest plane of the bilateral pedicles, and each section was parallel to the superior endplate of the vertebral body. A total of 4–5 TSAs for each BPS were simulated on the left and right sides of each lumbar vertebra (L1, L2, and L3: 0°, 5°, 10°, 15°; L4 and L5: 0°, 5°, 10°, 15°, 20°), passing through the midpoint of the pedicle stenosis. The distances from the anterior vertebral cortex (AVC) to the PGV (D_AVC-PGV_) were measured (Fig. 1). According to the D_AVC-PGV_, the PGV was classified into three types: close, distant, and noncontact PGVs. If the extension line of the BPS intersected the PGV and had a distance of less than 0.50 cm, “D_AVC-PGV < 0.50 cm_” represented a close PGV. If the extension of the BPS intersected the PGV and the distance was greater than or equal to 0.50 cm, “D_AVC-PGV ≥ 0.50 cm_” represented a distant PGV. If the extension line of the BPS did not intersect the PGV, it was expressed as “↑”, and “D_AVC-PGV↑_” represented a noncontact PGV. Second, the ratio of the three types of PGV at each TSA of the BPSs in each lumbar vertebra was calculated. The higher the ratio of the close PGVs, the higher the risk of potential injury to the PGV. The participants’ age, height, weight and body mass index for the three types of PGV at each TSA in every lumbar vertebra were collected. Two senior spine surgeons independently performed the data measurements, who had mastered the experimental methods used in this study and were skilled in completing the internal fixation of pedicle screws. In the measurement process, the CT images were magnified as much as possible to reduce measurement error. The authors had access to information that could identify individual participants during data collection. If the measurements differed by a value ≥0.30 cm, the measurements were taken again and submitted to a very senior spine specialist for judgment. We statistically analyzed two doctors’ measurements of the same parameters. If the differences were not significant between their data (*P* > 0.05), their averaged values were used for formal analysis to ensure the inter-rater reliability.

### Statistical analysis

Statistical processing was performed using SPSS19 software, and the measured anatomical parameters were expressed as $\overline{x}$ ± S. Comparing the left and right D_AVC-PGV ≥ 0.50 cm_, the data were normally distributed using an unpaired *t*-test, but the data were not normally distributed using the Mann-Whitney test. The constituent ratio of D_AVC-PGV↑_, D_AVC-PGV ≥ 0.50 cm_, and D_AVC-PGV < 0.50 cm_ at each TSA in L1-L5 was compared between the left and right sides using a two-tailed Fisher’s exact test. The samples’ age, height, weight and body mass index among the three types of PGVs at each TSA in every lumbar vertebra were compared using a one way ANOVA. Differences of *P* < 0.05 were considered statistically significant.

## Results

**Fig. 2:**
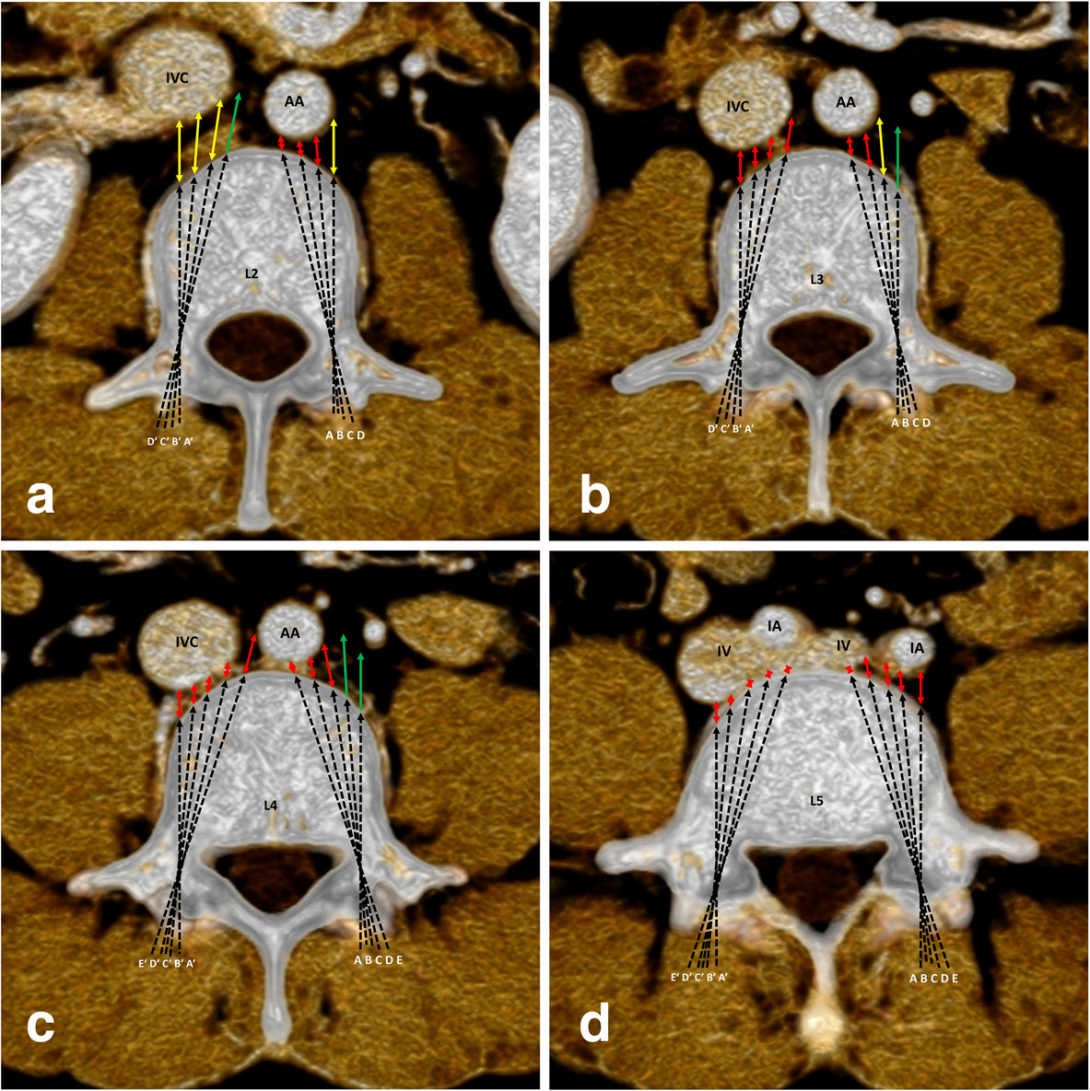
D_AVC-PGV_ at different TSAs in L2-L5. AA, abdominal aorta. IVC, inferior vena cava. IA, iliac artery. IV, iliac vein. A, B, C, D and E represent the left TSA of the BPS at 0°, 5°, 10°, 15°and 20°, respectively; A’, B′, C′, D’ and E’ represent the right TSA of the BPS at 0°, 5°, 10°, 15°and 20°, respectively

**Fig. 3:**
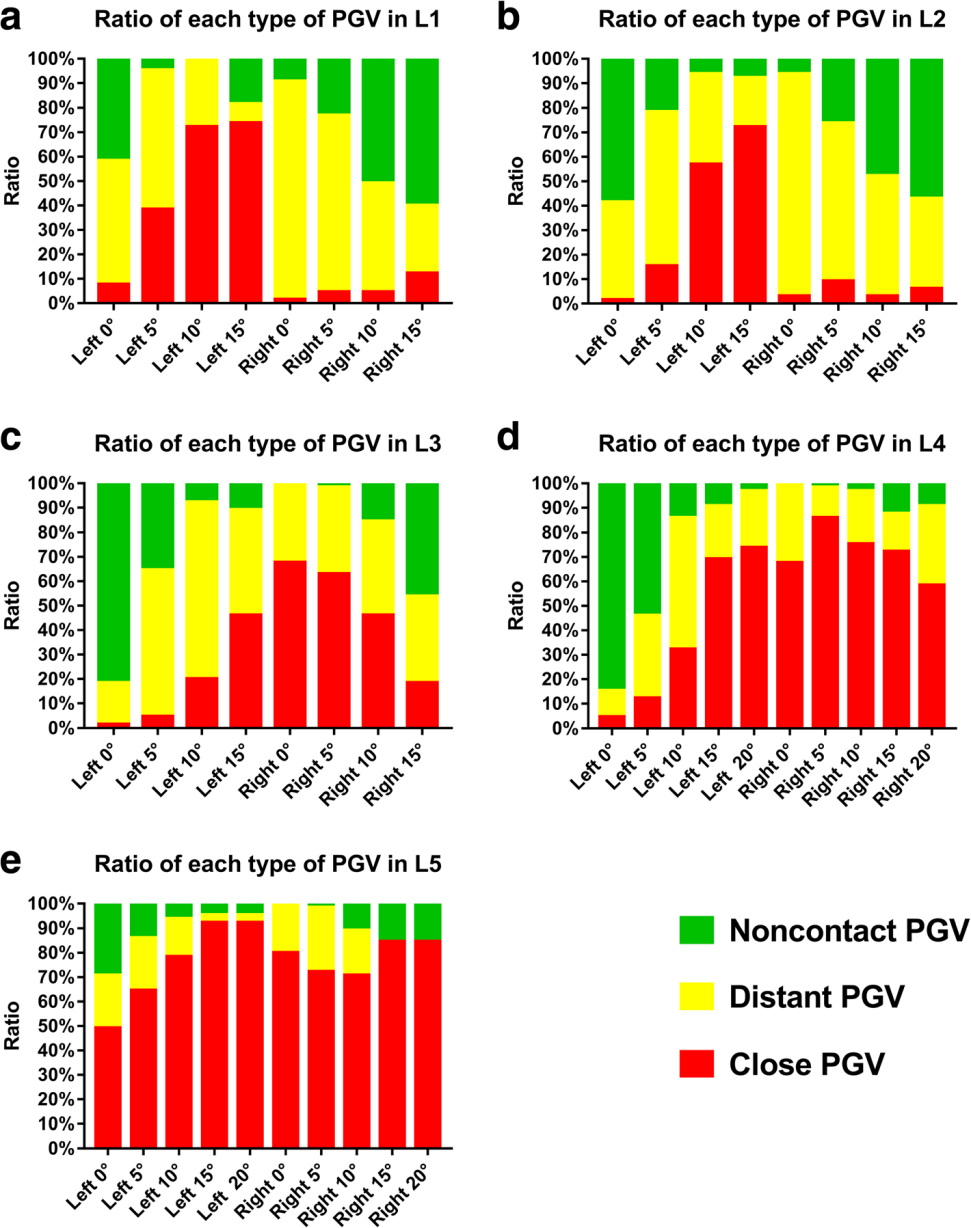
The ratios of three types of PGV at different TSAs in L1-L5. PGV, prevertebral great vessel

The participants comprised 34 males and 31 females aged 43.62 ± 9.48 years old (22–68 years old). The males were 44.32 ± 9.61 years old (22–68 years old), and the females were 42.84 ± 9.44 years old (24–61 years old). There was no significant difference in age between the males and females (*P* = 0.533). The D_AVC-PGV_ at each TSA of the BPSs in L1-L5 are shown in Table 1 and Fig. 2, and the ratios of the three types of PGVs at different TSAs are shown in Fig. 3.align="left" colname="c1" colnum="1"/>

**Table 1:** D_AVC-PGV_ at each TSA of the BPSs in L1-L5

Lumbar	TSA	Type of D_AVC-PGV_	Left	Right	*P*_1_	Left	Right	*P*_2_
			Number (%)	Number (%)		D_AVC-PGV_ (cm)	D_AVC-PGV_ (cm)	
L1	0°	↑	27 (41.54%)	6 (9.23%)				
		≥0.50 cm	33 (50.77%)	58 (89.23%)	0.000	0.87 ± 0.29	1.37 ± 0.39	0.000
		< 0.50 cm	5 (7.69%)	1 (1.54%)		0.30 ± 0.10	0.13	
L1	5°	↑	3 (4.62%)	15 (23.08%)				
		≥0.50 cm	37 (56.92%)	47 (72.31%)	0.000	0.80 ± 0.24	1.45 ± 0.37	0.000
		< 0.50 cm	25 (38.46%)	3 (4.62%)		0.30 ± 0.11	0.25 ± 0.17	
L1	10°	↑	0	33 (50.77%)				
		≥0.50 cm	18 (27.69%)	29 (44.62%)	0.000	0.64 ± 0.11	1.40 ± 0.48	0.000
		< 0.50 cm	47 (72.31%)	3 (4.62%)		0.33 ± 0.10	0.28 ± 0.11	
L1	15°	↑	12 (18.46%)	39 (60.00%)				
		≥0.50 cm	5 (7.69%)	18 (27.69%)	0.000	0.63 ± 0.08	0.88 ± 0.40	0.008
		< 0.50 cm	48 (73.85%)	8 (12.31%)		0.31 ± 0.09	0.36 ± 0.10	
L2	0°	↑	38 (58.46%)	4 (6.15%)				
		≥0.50 cm	26 (40.00%)	59 (90.77%)	0.000	1.05 ± 0.29	1.02 ± 0.38	0.731
		< 0.50 cm	1 (1.54%)	2 (3.08%)		0.31	0.45 ± 0.01	
L2	5°	↑	14 (21.54%)	17 (26.15%)				
		≥0.50 cm	41 (63.08%)	42 (64.62%)	0.535	0.78 ± 0.20	1.12 ± 0.42	0.000
		< 0.50 cm	10 (15.38%)	6 (9.23%)		0.34 ± 0.10	0.45 ± 0.03	
L2	10°	↑	4 (6.15%)	31 (47.69%)				
		≥0.50 cm	24 (36.92%)	32 (49.23%)	0.000	0.69 ± 0.19	0.92 ± 0.38	0.004
		< 0.50 cm	37 (56.92%)	2 (3.08%)		0.34 ± 0.08	0.44 ± 0.02	
L2	15°	↑	5 (7.69%)	37 (56.92%)				
		≥0.50 cm	13 (20.00%)	24 (36.92%)	0.000	0.65 ± 0.13	0.80 ± 0.20	0.025
		< 0.50 cm	47 (72.31%)	4 (6.15%)		0.32 ± 0.13	0.40 ± 0.06	
L3	0°	↑	53 (81.54%)	0				
		≥0.50 cm	11 (16.92%)	21 (32.31%)	0.000	1.28 ± 0.43	0.74 ± 0.18	0.000
		< 0.50 cm	1 (1.54%)	44 (67.69%)		0.34	0.41 ± 0.05	
L3	5°	↑	23 (35.38%)	1 (1.54%)				
		≥0.50 cm	39 (60.00%)	23 (35.38%)	0.000	0.99 ± 0.28	0.72 ± 0.17	0.000
		< 0.50 cm	3 (4.62%)	41 (63.08%)		0.37 ± 0.10	0.40 ± 0.08	
L3	10°	↑	5 (7.69%)	10 (15.38%)				
		≥0.50 cm	47 (72.31%)	25 (38.46%)	0.001	0.73 ± 0.19	0.76 ± 0.28	0.643
		< 0.50 cm	13 (20.00%)	30 (46.15%)		0.39 ± 0.06	0.35 ± 0.10	
L3	15°	↑	7 (10.77%)	30 (46.15%)				
		≥0.50 cm	28 (43.08%)	23 (35.38%)	0.000	0.60 ± 0.07	0.83 ± 0.21	0.000
		< 0.50 cm	30 (46.15%)	12 (18.46%)		0.37 ± 0.08	0.28 ± 0.12	
L4	0°	↑	55 (84.62%)	0				
		≥0.50 cm	7 (10.77%)	21 (32.31%)	0.000	0.93 ± 0.36	0.74 ± 0.20	0.089
		< 0.50 cm	3 (4.62%)	44 (67.69%)		0.26 ± 0.01	0.30 ± 0.13	
L4	5°	↑	35 (53.85%)	1 (1.54%)				
		≥0.50 cm	22 (33.85%)	8 (12.31%)	0.000	0.94 ± 0.28	0.58 ± 0.06	0.000
		< 0.50 cm	8 (12.31%)	56 (86.15%)		0.30 ± 0.15	0.21 ± 0.16	
L4	10°	↑	9 (13.85%)	2 (3.08%)				
		≥0.50 cm	35 (53.85%)	14 (21.54%)	0.000	0.79 ± 0.21	0.64 ± 0.15	0.014
		< 0.50 cm	21 (32.31%)	49 (75.38%)		0.32 ± 0.13	0.13 ± 0.14	
L4	15°	↑	6 (9.23%)	8 (12.31%)				
		≥0.50 cm	14 (21.54%)	10 (15.38%)	0.641	0.70 ± 0.24	0.81 ± 0.19	0.200
		< 0.50 cm	45 (69.23%)	47 (72.31%)		0.28 ± 0.13	0.14 ± 0.17	
L4	20°	↑	2 (3.08%)	6 (9.23%)				
		≥0.50 cm	15 (23.08%)	21 (32.31%)	0.127	0.69 ± 0.19	0.67 ± 0.14	0.819
		< 0.50 cm	48 (73.85%)	38 (58.46%)		0.28 ± 0.14	0.12 ± 0.17	
L5	0°	↑	19 (29.23%)	0				
		≥0.50 cm	14 (21.54%)	13 (20.00%)	0.000	0.79 ± 0.21	0.79 ± 0.22	0.950
		< 0.50 cm	32 (49.23%)	52 (80.00%)		0.08 ± 0.16	0.16 ± 0.18	
L5	5°	↑	9 (13.85%)	1 (1.54%)				
		≥0.50 cm	14 (21.54%)	17 (26.15%)	0.035	0.86 ± 0.32	0.70 ± 0.11	0.098
		< 0.50 cm	42 (64.62%)	47 (72.31%)		0.08 ± 0.15	0.07 ± 0.13	
L5	10°	↑	4 (6.15%)	7 (10.77%)				
		≥0.50 cm	10 (15.38%)	12 (18.46%)	0.525	0.69 ± 0.11	0.58 ± 0.08	0.011
		< 0.50 cm	51 (78.46%)	46 (70.77%)		0.08 ± 0.15	0.03 ± 0.09	
L5	15°	↑	3 (4.62%)	10 (15.38%)				
		≥0.50 cm	2 (3.08%)	0	0.046	0.69 ± 0.13		
		< 0.50 cm	60 (92.31%)	55 (84.62%)		0.08 ± 0.15	0.07 ± 0.14	
L5	20°	↑	3 (4.62%)	10 (15.38%)				
		≥0.50 cm	2 (3.08%)	0	0.046	0.58 ± 0.05		
		< 0.50 cm	60 (92.31%)	55 (84.62%)		0.04 ± 0.10	0.08 ± 0.16	

*P*_1_: Two-tailed Fisher’s exact test with a constituent ratio of D_AVC-PGV↑_, D_AVC-PGV ≥ 0.50 cm_, and D_AVC-PGV < 0.50 cm_ between the left and right sides at each TSA. *P*_2_: Unpaired *t*-test, left D_AVC-PGV ≥ 0.50 cm_ vs. rightD_AVC-PGV ≥ 0.50 cm_

In L1, there were significant differences in the constituent ratios of D_AVC-PGV↑_, D_AVC-PGV ≥ 0.50 cm_, and D_AVC-PGV < 0.50 cm_ between the left and right sides at 0°, 5°, 10°, and 15°, respectively (*P* < 0.001). The left D_AVC-PGV ≥ 0.50 cm_ was 0.63 cm–0.87 cm, and the right D_AVC-PGV ≥ 0.50 cm_ was 0.88 cm–1.37 cm. The right D_AVC-PGV ≥ 0.50 cm_ was larger than the left, and the differences were significant (*P* < 0.01). The lowest ratio of the close PGVs on the left side of L1 was 7.69% at 0°, followed by 38.46% at 5°, and the highest was 73.85% at 15°. The ratios of the close PGVs on the right side of L1 were 1.54, 4.62, 4.62 and 12.31% at 0°, 5°, 10°, and 15°, respectively.

In L2, there were significant differences in the constituent ratios of D_AVC-PGV↑_, D_AVC-PGV ≥ 0.50 cm_, and D_AVC-PGV < 0.50 cm_ between the left and right sides at 0°, 10°, and 15°, respectively (*P* < 0.001), but there were no significant differences at 5° (*P* > 0.05). The right D_AVC-PGV ≥ 0.50 cm_ at 5°, 10°, and 15° in L2 were larger than those on the left, and the differences were significant (*P* < 0.05). The left D_AVC-PGV ≥ 0.50 cm_ was 1.05 ± 0.29 cm at 0° in L2, and the right was 1.02 ± 0.38 cm (*P* > 0.05). The lowest ratio of the close PGVs on the left side of L2 was 1.54% at 0° followed by 15.38% at 5°, and the highest was 72.31% at 15°. The ratios of the close PGVs on the right side of L2 were 3.08, 6.15, 6.15 and 9.23% at 0°, 5°, 10°, and 15°, respectively.

In L3, the constituent ratios of D_AVC-PGV↑_, D_AVC-PGV ≥ 0.50 cm_, and D_AVC-PGV < 0.50 cm_ between the left and right sides were significantly different at 0°, 5°, 10° and 15°, respectively (*P* < 0.001). The left D_AVC-PGV ≥ 0.50 cm_s at 0° and 5° in L3 were larger than those on the right side, and the differences were significant (*P* < 0.001). The lowest ratio of the close PGVs on the left side of L3 was 1.54% at 0°, followed by 4.62% at 5°, and the highest was 46.15% at 15°. The lowest ratio of the close PGVs on the right side of L3 was 18.46% at 15°, followed by 46.15% at 10°, and the highest was 67.69% at 0°.

In L4, the constituent ratios of D_AVC-PGV↑_, D_AVC-PGV ≥ 0.50 cm_, and D_AVC-PGV < 0.50 cm_ between the left and right sides were significantly different at 15° and 20°, respectively (*P* < 0.001), but the constituent ratios were not significantly different at 0°, 5°, and 10°, respectively (*P* > 0.05). The lowest ratio of the close PGVs on the left side of L4 was 4.62% at 0°, followed by 12.31% at 5°, and the highest ratio was 73.85% at 20°. The lowest ratio of the close PGVs on the right side of L4 reached 58.46% at 20°.

In L5, the constituent ratios of D_AVC-PGV↑_, D_AVC-PGV ≥ 0.50 cm_, and D_AVC-PGV < 0.50 cm_ between the left and right sides were significantly different at 0°, 5°, 15° and 20°, respectively (*P* < 0.05), and the number of D_AVC-PGV < 0.50 cm_ was the highest. The lowest ratio of the close PGVs on the left side of L5 reached 49.23% at 0°, and the lowest ratio of the close PGVs on the right side of L5 reached 70.77% at 10°.

There were no significant differences in the participants’ age, height, weight and body mass index among the three types of PGV at each TSA in every lumbar vertebra (*P* > 0.05). Close PGV represented a narrow avascular space in front of the vertebral body that was no greater than 0.5 cm, so BPS insertion carried a high risk of injury to the PGV. The lower the ratio of the close PGVs, the lower the risk of potential injury to the PGV. Generally, the ratio of close PGVs should be limited to 10% or less. Based on the above results, the following TSAs of BPS in L1-L5 were recommended as follows (Table 2). The recommended left TSA of PBS in L1 was 0°, and the right TSA was 0°-10°; the recommended left TSA of PBS in L2 was 0°, and the right TSA was 0°-15°; the recommended left TSA of PBS in L3 was 0°-5°; the recommended left TSA of PBS in L4 was 0°; BPS was not recommended for the right side of L3, the right side of L4, or either side of L5.colspec align="left" colname="c1" colnum="1"/>

**Table 2:** The recommended TSA of the BPSs in L1-L5

Lumbar vertebra	Left					Right				
	0°	5°	10°	15°	20°	0°	5°	10°	15°	20°
L1	R	×	×	×		R	R	R	×	
L2	R	×	×	×		R	R	R	R	
L3	R	R	×	×		×	×	×	×	
L4	R	×	×	×	×	×	×	×	×	×
L5	×	×	×	×	×	×	×	×	×	×

Note: R represents recommendation; × represents no recommendation

## Discussion

The BPS technique was first reported for S1. In 1991, Mirkovic et al. [19] researched the risk of vascular, nervous and visceral injuries in front of a pedicle screw in S1 and determined the safe area for BPSs in S1. In 2000, Zhu et al. [20] confirmed the mechanical advantages of BPSs in S1. In recent years, a large number of clinical studies have confirmed the feasibility of BPSs and tricortical pedicle screws in S1 [21, 22]. In 2011, Ponnusamy et al. [11] proposed that the lumbar and thoracic vertebrae could be fixed with a BPS but suggested that doctors should be aware of possible injury to the PGV. In 2012, Bezer et al. [16] confirmed that BPSs were more resistant to direct vertebral rotation during scoliosis orthopedic surgery, which was especially suitable for the convex side of scoliosis. In 2015, Le Cann et al. [23] performed a mechanical experiment with BPSs using pig lumbar vertebrae and confirmed that BPSs could be beneficial in surgery for adolescent scoliosis. Karami et al. [15] performed a mechanical experiment using osteoporotic cadaveric lumbar vertebrae. Three groups were classified according to their screw insertion depth: midbody, pericortical and bicortical, of which the pullout force and energy were 583 ± 306 N and 1.75 ± 1.98 N·m, 713 ± 321 N and 2.40 ± 1.79 N·m, and 797 ± 285 N and 2.97 ± 2.33 N·m, respectively (*P* < 0.05). It was concluded that the additional purchase of the stiff anterior cortex was indispensable for achieving superior stability and stiffness of the screw-bone interface. The mechanical strength of BPS fixation has been confirmed with methods ranging from animal experiments to cadaveric experiments and scoliosis surgery. However, there is still a lack of reports on its clinical application. The main reason for this is that spine surgeons are wary of the potential damage to the PGVs.

Currently, damage to the PGVs from pedicle screws in the thoracic vertebrae has been reported in several papers [24–28], but this damage has rarely been reported in the lumbar vertebrae [18]. In 2010, Foxx et al. [29] retrospectively analyzed 182 patients with thoracolumbar and lumbosacral pedicle screw fusion. A total of 680 pedicle screws were placed, 33 of which were in contact with the great vessels, including 4 cases of the aorta, 7 cases of the iliac artery, and 22 cases of the iliac vein. No patients developed any symptoms or sequelae due to contact between the great vessels and pedicle screws during the 44-month (range 5–109 months) follow-up period. Their conclusion was that in general, the position of screws that contacted the great blood vessels and did not cause any symptoms should not be changed, but this decision must be weighed against the relative risk of leaving the screw in place. In the above example, the anterior vertebral cortex was unintentionally penetrated. If the cortex was cautiously engaged and the screw penetrated with the tip to the appropriate length, the structures in front of the vertebral body would not be in danger [16]. BPSs were not as terrible as previously imagined, and this procedure was not impossible to perform.

In the lumbar vertebrae, there is a certain distance between the anterior vertebral cortex and the abdominal aorta, inferior vena cava, and iliac vessels that provides for the feasibility of lumbar BPS fixation. Di Silvestre et al. [30] left screws with a lateral cortical penetration that were at least 5 mm in place during a mean follow-up period of 4 years, and they observed no symptoms in these cases. Bezer et al. [16] suggested that the BPSs should penetrate with the tip no more than 1 thread beyond the cortical surface, and Karami et al. [15] proposed that the screw tip should penetrate no more than 2 mm through the anterior cortex of the vertebral body. Combined with these reports and considering the PGVs might be irritated due to their pulsating, the appropriate safe distance between the PBS and the PGV was approximately 5 mm. Since the L4 and L5 vertebral bodies are wider, L4 and L5 were studied at five TSAs. This study found that a BPS could not be placed in every lumbar vertebra. The right side of L3 and L4 and both sides of L5 are not recommended for BPSs because they are closer to a PGV (18.46–92.31%), given the lower inferior vena cava and the iliac vessels’ proximity to the lower lumbar vertebrae [31]. Generally, with mastery of the basic implantation technique of the pedicle screw and with the help of the fluoroscopy machine, it was unlikely for the BPS to exceed 5 mm in front of the vertebral body. Therefore, when the D_AVC-PGV_ was greater than 5 mm, the BPS was not likely to damage the PGVs. However, if the D_AVC-PGV_ was less than 5 mm, the BPS had a higher risk of injuring the PGVs due to the narrow avascular space in front of the vertebral body. The smaller the D_AVC-PGV_, the higher the risk of injury to the PGV. From the anatomical perspective of the PGVs, in L1, the recommended left TSA of the BPS was 0°, and the right TSA was 0–10°; in L2, the recommended left TSA of the BPS was 0°, and the right TSA was 0–15°; in L3, the recommended left TSA of the BPS was 0–5°; in L4, the recommended left TSA of the BPS was 0°. At 0° TSA, the screw was placed vertically, where the tip was closer to the lateral side of the vertebral body.

Some suggestions for avoiding damage to the PGVs when placing a BPS are as follows. First, the BPS insertion methods that are recommended in this study do not completely avoid injury to the PGV. The individual principle should be encouraged. Each patient should have a routine abdominal CTA examination before surgery, and the surgical strategy should be planned in detail to confirm the angle and depth of the BPS. Second, high-precision equipment should be used during surgery. Balling et al. [32] reported that they placed pedicle screws with the help of 3D-fluoroscopy navigation, and the correct rate of pedicle screw depth was 96.4%. Intraoperative navigation accurately controlled the TSA of the pedicle screw, helping to confirm the position of the screw tip. Without the assistance of navigation systems, the minor angular differences (5 degrees) in the insertion of BPS were very difficult to achieve with good precision. Finally, the BPS broke through the anterior cortex of the lumbar vertebrae by no more than 2 threads, using 2.5 mm increments in screw length to make the procedure safer [16].

This study also had certain limitations, such as a small sample size and unavoidable measurement error.

## Conclusion

From the anatomical perspective of the PGVs, the BPSs were not suitable for insertion into every lumbar vertebra. Furthermore, the recommended insertion methods for the BPSs in L1-L4 were different.

## Data Availability

Readers can access the data and material supporting the conclusions of the study by contacting Liehua Liu at 651520561@qq.com.

## Abbreviations

AVC: Anterior vertebral cortex
BPS: Bicortical pedicle screw
CTA: Computed tomography angiography
D_AVC-PGV_: Distances from the AVC to the PGV
PGV: Prevertebral great vessel
TSA: Transverse screw angle
